# Unravelling the Role of Trophoblastic-Derived Extracellular Vesicles in Regulatory T Cell Differentiation

**DOI:** 10.3390/ijms20143457

**Published:** 2019-07-14

**Authors:** Árpád Ferenc Kovács, Nóra Fekete, Lilla Turiák, András Ács, László Kőhidai, Edit I. Buzás, Éva Pállinger

**Affiliations:** 1 Department of Genetics, Cell- and Immunobiology, Semmelweis University, H-1085 Budapest, Hungary; 2 MS Proteomics Research Group, Research Centre for Natural Sciences, Hungarian Academy of Sciences, H-1051 Budapest, Hungary; 3 MTA-SE Immune-Proteogenomics Extracellular Vesicle Research Group, H-1085 Budapest, Hungary; 4 HCEMM-SE Extracellular Vesicle Research Group, H-1085 Budapest, Hungary

**Keywords:** extracellular vesicles, EVs, HSPE1, miRNA, C19MC, regulatory T cells

## Abstract

Regulatory T cells (T_reg_) are mandatory elements in the maintenance of human pregnancy, but their de novo differentiation has not been completely exposed. HSPE1 chaperone expressing trophoblast cells may have a role in it. Trophoblast-derived extracellular vesicles (EVs), either at the feto–maternal interface or in circulation, target CD4^+^ T cells. We hypothesized that HSPE1-associated trophoblastic cell line (BeWo)-derived EVs are active mediators of T_reg_ cell differentiation. We proved at first that recombinant HSPE1 promote human T_reg_ cell differentiation in vitro. Developing a CRISPR-Cas9 based *HSPE1* knockout BeWo cell line we could also demonstrate, that EV-associated HSPE1 induces T_reg_ development. Next-generation sequencing of miRNA cargo of BeWo-EVs characterized the regulatory processes of T_reg_ polarization. By the use of single-cell transcriptomics analysis, seven T_reg_ cell subtypes were distinguished and we demonstrated for the first time that the expression level of *HSPE1* was T_reg_ subtype dependent, and *CAPG* expression is characteristic to memory phenotype of T cells. Our data indicate that *HSPE1* and *CAPG* may be used as markers for identification of T_reg_ subtypes. Our results suggest, that trophoblastic-derived iEVs-associated HSPE1 and miRNA cargo have an important role in T_reg_ cell expansion in vitro and *HSPE1* is a useful marker of T_reg_ subtype characterization.

## 1. Introduction

During human pregnancy the maternal immune system has to fine balance itself, in order to develop and maintain immune tolerance toward the semi-allograft fetus. An ongoing equilibrium between proinflammatory and anti-inflammatory stimuli characterize the immune system, and a tolerogenic environment is essential for the maintenance of pregnancy [1,2]. Immune suppressive T_reg_ cells are crucial players of peripheral tolerance [3]. T_reg_ cells are found at high levels in decidual tissues, and recently it has been described that in vitro co-culture of extravillous trophoblast cells with blood derived CD4^+^ T cells induces T_reg_ cell differentiation [4]. Nevertheless, it has not been clearly demonstrated what mechanisms are involved in the de novo differentiation of T_reg_ cells at the feto–maternal interface [5,6]. Although the generation of peripheral T_reg_ cells is less clearly understood [7], intercellular communication may have an important role in it. [8,9]. One of the most dynamic forms of intercellular communication is mediated by extracellular vesicles (EVs) [10]. EVs released by cells are heterogeneous in size, the two most well-studied subtypes are the intermediate-sized EVs (size range 150–800 nm, iEVs) and small EVs (size range 35–150 nm, sEVs) [11]. The placenta is an abundant and transient source of EVs [12]. On the other hand, the key feature of the protective environment is particularly realized at the placental level via the establishment and maintenance of tolerance.

Trophoblast cells highly express the HSPE1 protein [13]. The main annotated role of HSPE1 is the chaperonin mediated protein folding together with Hsp60 [14]. It has also been suggested that HSPE1 may be critical in the suppression of T cell activation [15]. In myocytes, it has been also described that HSPE1 inhibits the proapoptotic activity of cells and shifts the cell fate balance toward survival [16]. It has also been proposed that HSPE1 is selectively released from proliferating cells and is an active player of cell signalling network [15]. Recombinant HSPE1 selectively binds to human CD4^+^ T cells in vitro [17] and can induce T_reg_ differentiation in mouse animal model [18]. Besides proteins, miRNAs are the most important molecular cargo of EVs [19]. miRNAs dynamically regulate the expression of gene networks (1) and contribute to the function of cell differentiation at the posttranscriptional level [20]. EV-associated miRNome is regarded as one of the most promising clinical biomarker for diagnosis, prognosis, and therapeutic options [21]. On the basis of these data, we hypothesized that trophoblast-derived EV-associated HSPE1 and miRNA cargo specify the generation and heterogeneity of T_reg_ cells at the feto–maternal interface.

## 2. Results

### 2.1. miRNA and Protein Pattern of BeWo-Derived EVs

**Figure 1:**
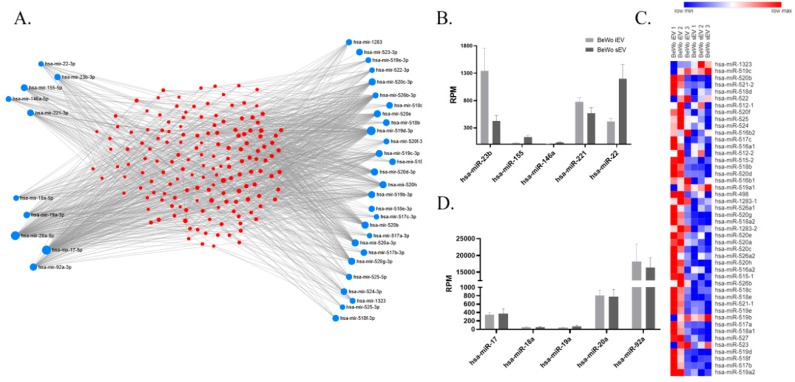
miRNA content of trophoblastic-derived EVs. (**A**) Overview of miRNAs found in trophoblastic (BeWo cells)-derived EVs and their cell differentiation-associated target genes. In the upper left miRNAs involved in the immunological tolerance are displayed. In the lower left, the miR17-92 cluster and, on the right, the placental-specific C19MC cluster are showed. Red dots mark the target genes of the miRNAs. (**B**) Expression of miRNAs involved in immunological tolerance (expression is given in reads per million (RPM), *n* = 3) (**C**) Expression of miRNAs on the C19MC miRNA cluster, showing that most of the miRNAs are showing a higher expression in the iEV fraction. (**D**) Expression of miR17-92 cluster (expression is given in reads per million (RPM), *n* = 3).

Altogether we detected 255 common miRNAs by next generation sequencing in BeWo-derived iEVs and sEVs. There were 111 unique miRNAs to iEV fraction and 20 miRNAs in the sEV fraction, however none of these miRNAs are known to have immunoregulatory functions. Therefore, we focused on the common miRNA pattern. All 46 miRNA gene transcripts of the trophoblast-specifically expressed C19MC cluster were detected in both iEV and sEV fraction miRNA pattern (Figure 1A, C, *n* = 3). Hsa-miR-23b is also expressed in EVs, which inhibits the Th17 signalling. Hsa-miR-146a and hsa-miR-155 which are critical in T_reg_ cells were found in the EV fractions. Hsa-miR-22 and hsa-miR-221, known as tolerance-associated miRNAs were highly expressed in EVs (Figure 1A,B). All members of the hsa-miR-17-92 polycistronic miRNA cluster, of critical value in differentiation of antigen-specific IL-10 producing T_reg_ cells were detectable in EVs (Figure 1A,D).

**Figure 2:**
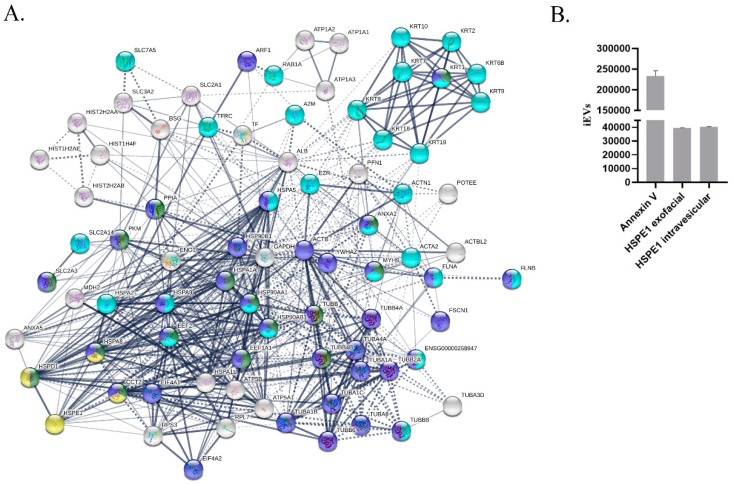
HSPE1 content of BeWo iEVs. (**A**) Protein interaction network of proteins found in Bewo-derived iEVs. Dark blue color represents the proteins involved in immune system processes, light blue color marks the proteins involved in leukocyte activation, and the proteins playing a role in protein folding (k-mean clustering) are indicated in yellow. (**B**) FACS-based validation of HSPE1 association with BeWo-derived iEVs.

We identified by mass spectrometry 81 proteins in iEV and 31 proteins in the sEV fraction. We found, in the iEV fraction, 27 proteins related to immune system process (GO:0002376, *p* = 2.09 × 10^−5^), out of these proteins 16 are associated with leukocyte activation (GO:0045321, *p* = 2.89 × 10^−5^) and 29 proteins associated with cell differentiation (GO:0030154, *p* = 0.0013). De novo protein folding protein, HSPE1 (GO:0006458, *p* = 0.00072) was also identified in the iEV samples (Figure 2A). The presence of HSPE1 was validated by flow cytometry and it was detected both on the exofacial surface and in the intra-vesicular compartment of iEVs (Figure 2B). HSPE1 was unique to the iEV fraction, it could not be detected in sEVs (Supplementary Figure S1).

### 2.2. Recombinant HSPE1 (rHSPE1) and iEVs Induce Human T_reg_ Cell Expansion In Vitro

**Figure 3:**
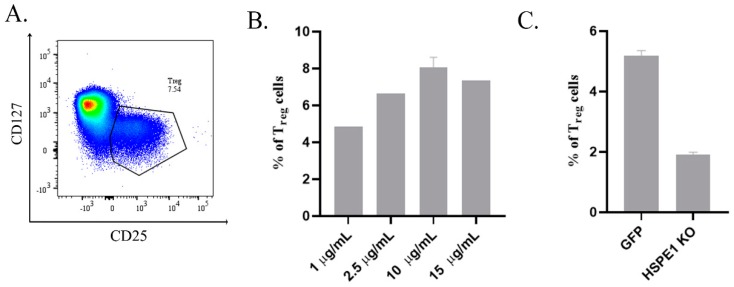
rHSPE1, BeWo GFP-iEV, and BeWo HSPE1 KO-iEV induced T_reg_ differentiation from CD4^+^ Th cells. (**A**) Representative FACS dot plot showing the expanded T_reg_ cell population (defined as CD25^+^CD127^lo^) upon rHSPE1 treatment (*x*-axis showing the expression of CD25, *y*-axis showing the expression of CD127). (**B**) Concentration dependent T_reg_ cell expansion upon rHSPE1 treatment. (**C**) T_reg_ cell expansion upon GFP control transfected-derived iEVs (abbreviated as GFP) and HSPE1 KO-derived iEVs (abbreviated as HSPE1-KO).

rHSPE1 induced CD25^+^CD127^lo^ T_reg_ cell expansion from human CD4^+^ T cells. We found that 10 µg/ mL of rHSPE1 is the most potent concentration for in vitro T_reg_ cell expansion (rHSPE1 8.07 ± 0.53 % vs. untreated 1.98 ± 0.02%) (Figure 3A,B). In vitro generated CD25^+^CD127^lo^ T_reg_ cells were sorted and showed viability by having positive migratory and motility capacity for 3 h under holomicroscopic analysis (Supplementary Figure S2).

To decide whether HSPE1 induced T_reg_ generation is associated with iEVs, we have developed an HSPE1-KO BeWo cell line construction. We transfected the BeWo cells with either GFP control plasmid or HSPE1 targeting sgRNA. Transfection efficiency was validated with Sanger sequencing and qPCR (Supplementary Figure S3). iEVs isolated from GFP transfected BeWo cells induced T_reg_ cell expansion comparable to the rHSPE1 protein (5.36%), however iEVs isolated from HSPE1 KO BeWo cells caused a similar effect as untreated control (1.85%) (Figure 3C). 

### 2.3. HSPE1 is Differentially Expressed in the T_reg_ Subtypes

**Figure 4:**
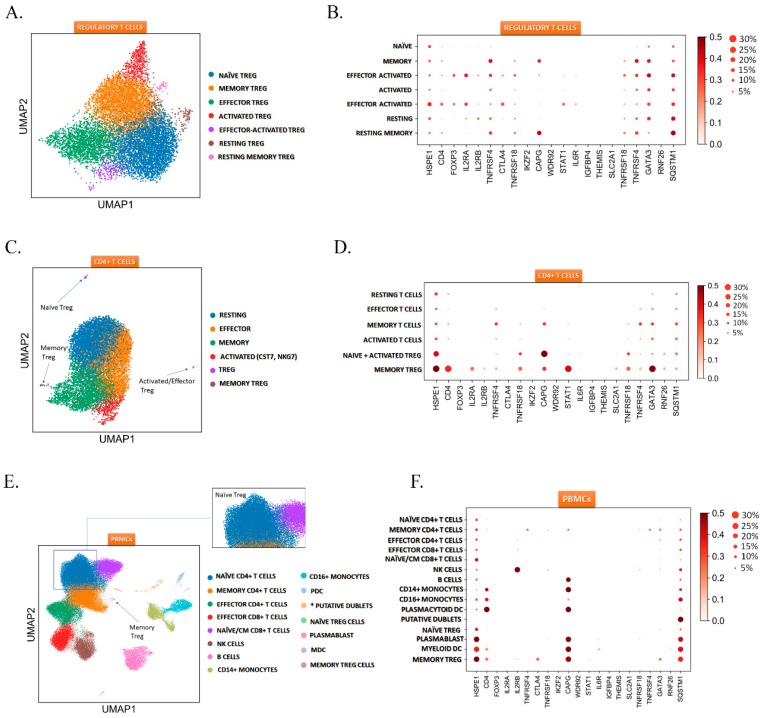
Regulatory T cell heterogeneity revealed by single cell transcriptomics. (**A**) UMAP clustering of T_reg_ cells subsets. (**B**) T_reg_ cell subtype dependent expression of *HSPE1*. (**C**) UMAP clustering of CD4^+^ T cell subsets, blue arrows showing identified T_reg_ cell subsets. (**D**) CD4^+^ T cell subsets dependent expression of *HSPE1.* (**E**) UMAP clustering of PBMCs, blue arrow showing memory T_reg_ cell population and green points showing naïve T_reg_ cell population dispersed in CD4^+^ naïve cells. (**F**) PBMCs subsets dependent expression of *HSPE1.*

To address the whether HSPE1 plays a role in the maintenance of T_reg_ cells we used of single-cell transcriptomic approach. The analysis showed that all seven T_reg_ cell subtypes expressed the *HSPE1* among CD4^+^CD25^+^ T_reg_ cells. *HSPE1* showed a cluster dependent expression (Figure 4A,B). To compare how does the expression of HSPE1 observed in T_reg_ cells relate to CD4^+^ cells and peripheral blood mononuclear cells (PBMCs) we applied the marker genes identified in the T_reg_ single-cell data to CD4^+^ T cells and could successfully differentiate three T_reg_ cell subtypes in this dataset: naïve, activated/effector, and memory T_reg_ cells (Figure 4C,D).

Furthermore, we went on and successfully identified two T_reg_ cell clusters in PBMCs: naïve T_reg_ and memory T_reg_ cells based on the subtype-specific marker genes identified in the CD4^+^CD25^+^ T_reg_ cells. With our approach, we could also identify the naïve T_reg_ cells among naïve T cells (Figure 4E,F). In all three datasets, the *HSPE1* expression was T_reg_ cell dependent, and we also identified the *CAPG* expression as a memory T_reg_ and memory T cell-specific among T cells, suggesting the expression of *HSPE1* and *CAPG* as possible T_reg_ subtype markers. To summarize, the single-cell transcriptomic analysis suggests that HSPE1 may have a maintenance role in T_reg_ cells, however this hypothesis needs to be confirmed by further downstream analysis.

## 3. Discussion

Regulatory T cells play a central role in the induction and maintenance of maternal immune tolerance during pregnancy. T_reg_ cells inhibit (1) the proliferation and cytokine production in both effector CD4^+^ and CD8^+^ T cells, (2) the immunoglobulin production by B cells, (3) the cytotoxic activity of natural killer (NK) cells, and (4) the maturation of dendritic cells (DC) [22]. miRNAs are small non-coding RNA molecules which are potent regulators of gene networks. They are released into the extracellular space and systemic circulation, contributing to tissue homeostasis and disease pathophysiology. They may be packaged into EVs or may bind to proteins including argonaute-2 (Ago2), nucleophosmin1, or high-density lipoproteins. The largest cluster of miRNAs in the human genome is the chromosome 19 miRNA cluster (C19MC) which is highly expressed in the human placenta. The rapid decline of C19MC miRNAs after delivery has been described [23]. Among all human placental miRNAs, the members of the C19MC cluster have the highest expression levels [24]. C19MC cluster is also expressed in trophoblastic placental cell lines, including the choriocarcinoma cell lines JEG3, JAr, and BeWo. We demonstrated, that all members of the C19MC cluster are expressed in BeWo-derived EVs and their expression levels were the highest among BeWo-EV-associated miRNAs. We could also identify the presence of hsa-miR-221 and hsa-miR22, which are tolerance-associated miRNAs, through silencing of inflammatory genes and chromatin remodelling. We could also determine the presence of hsa-miR-155, that is highly expressed in T_reg_ cells and plays a role in T_reg_ cell proliferation via targeting SOCS1 [25]. The presence of miR-17-92 polycistronic miRNA cluster (hsa-miR 17, 18a, 19a, 20a, and 92) also confirmed the effects of BeWo-iEVs in T_reg_ differentiation because of it is an indispensable influence in antigen-specific IL-10 production of T_reg_ cells. [1]. miRNAs that play an important role in T_reg_ cell functions were also revealed, including hsa-miR146a, which is necessary for T_reg_ mediated suppression [26] and hsa-miR-23b, which is a competent suppressor of Th17 signalling. 

We have identified by mass spectrometry the presence of HSPE1 protein and confirmed the presence of HSPE1 both on the surface of iEVs and in the vesicular lumen by a flow cytometric approach. The presence of HSPE1 has been reported in B-cell derived sEVs [27] and cancer cell-derived EVs [28,29,30] by mass spectrometric analysis, but the function of EV-associated HSPE1 has not been evaluated. In our proteomic analysis, we have identified proteins involved in the biogenesis of iEVs (enriched in microvesicles), including annexins (ANXA2, ANXA5), Rab proteins (RAB1A), and ADP-ribosylation factors (ARF1). 

As it was previously reported recombinant mouse HSPE1 induces regulatory T cell differentiation in vitro [18]. In our experiments, we investigated this phenomenon in human experimental systems [17]. We proved that human rHSPE1 induced T_reg_ cell differentiation in vitro too and trophoblastic iEVs associated HSPE1 could also mediate this effect as was confirmed by the using of HSPE1 KO cell line. Understanding the molecular mechanisms that provide the necessary signals for a continuously regulated active immune cell compartment may pave the way to unravel the highly and tightly controlled molecular cascade-network events and provide new biomarkers and potent therapies for the altered molecular event-induced pregnancy-specific disorders.

## 4. Materials and Methods 

### 4.1. Peripheral Blood Collection

Peripheral venous blood collected in 6mL Vacutainer^®^ EDTA of Becton Dickinson (BD San Jose, California, USA) from healthy non-pregnant donors (*n* = 10) was obtained from the Hungarian National Blood Transfusion Service. The study was approved by the Scientific and Research Ethics Commission (TUKEB) of Hungary (Approval code: 4834-0/2010-1018EKU; Date of approval: 18.10.2010). Donor-informed consent was signed by each donor and the guidelines and regulations of the Helsinki Declaration in 1975 were followed. Peripheral blood mononuclear cells (PBMCs) were separated using Histopaque^®^-1077 (Sigma, St. Louis, MO, USA), as a density gradient cell separation medium and stored at −80 °C until use.

### 4.2. Culturing of BeWo Cells

BeWo cell line (ATCC^®^ CCL-98™) was obtained from American Type Culture Collection. The cells were cultured in Ham’s F12/K medium supplemented with 2 mM L-glutamine, 10% GIBCO^®^ FBS (Thermo Fisher Scientific, Waltham, MA, USA), 5% glucose, 1.0 mM sodium pyruvate, 1% of Gibco^®^ MEM Non-Essential Amino Acids, 100 U/mL penicillin and 100 µg/mL streptomycin (Thermo Fisher Scientific, Waltham, MA, USA). Cells were maintained as monolayers in 25 or 75 cm^2^ flasks at 37 °C in the humidified atmosphere, in the presence of 5% CO2. All chemical reagents were purchased from Sigma-Aldrich Company (St. Louis, MO, USA).

### 4.3. Generation of HSPE1 Knockout (HSPE1 KO) BeWo Cells

CRISPR-Cas9 approach was applied to generate the HSPE1 KO BeWo cell line. First, to optimize the electroporation conditions, we used the Cell Line Optimization 4D-NucleofectorTM X Kit (Lonza, Cologne, Germany) according to the manufacturer’s instructions. Briefly, BeWo cells were centrifuged at 90 *g* for 10 min at room temperature (RT) and the medium was discarded completely. Cell pellets were resuspended in a cocktail containing 82% Solution SF (or SE or SG) and 18% Supplement obtaining a cell density of 1.8 × 10^5^ BeWo cells/ 19.6 µL. 0.4 µg of pMaxGFP plasmid vector (1 µg/mL) per 1.8 × 10^5^ BeWo cells was added to the cell suspension. Next, we homogenized the cell suspension by pipetting up-and-down and added 20 µL of BeWo cells to each well of the nucleocuvette. We ran the cell line optimization programs within the Nucleofector 4D-X. After nucleofection, we incubated the cells for 10 min at RT. After the incubation period, we added 30 µL 20% FBS containing RPMI-1640 media and incubated the cells for another 10 min at RT. Cells were transferred to 24 well Tc treated culture plates (Eppendorf AG, Hamburg Germany) and were analyzed by fluorescent microscopy and FACS for analysing of GFP signal (success of transfection) and cell viability (assessed by propidium iodide incorporation by FACS). Based on the optimization data the Supplement SF and transfection program EN-150 was chosen (viability 85%, transfection success 90%, Supplementary Figure S3). Next synthetic guide RNAs (sgRNA) were designed to target the *HSPE1* gene using Synthego CRISPR Design Tool (sgRNA-1 G*C*U*GCUGAAACUGUAACCAA and sgRNA-2 U*A*A*ACGCUUGUCCUGCCUGU) (Synthego Corporation, California, USA, Supplementary Figure S4). sgRNAs were rehydrated in 1x TE buffer obtaining a concentration of 100 pmol/µL) and the same transfection protocol was used as described above, with the addition of 20 pmol Cas9 nuclease 2NLS, S. pyogenes (Synthego Corporation, California, USA)/ well along with sgRNAs. Viability of GFP and HSPE1 KO cells was validated by FACS.

### 4.4. Validation of HSPE1 Knockout (HSPE1 KO) BeWo Cells

The transfection efficiency was evaluated and validated by several methods. DNA was isolated from GFP and HSPE1 KO BeWo cells with Blood/Cell DNA Mini Kit (Geneaid Biotech Ltd., New Taipei, Taiwan) and the concentration was determined with Qubit 4 fluorometer using the Qubit 1x dsDNA HS Assay Kit (Thermo Fisher Scientific, Waltham, MA, USA). Sanger sequencing was used to confirm the indels produced by HSPE1 specific sgRNA-2. Sequencing data were analyzed with Seq Scanner v2 (Supplementary Figure S4D). The Sanger sequencing data was used for ICE analysis to detect the indel frequencies and to determine the effectiveness of the transfection reaction (Supplementary Figure S4C). For gene expression analysis, first total RNA was isolated with RNeasy Mini Kit (Qiagen, Venlo, The Netherlands) from GFP and HSPE1 KO BeWo cells. The RNA concentration was determined with Qubit 4 fluorometer using the Qubit™ RNA HS Assay Kit (Thermo Fisher Scientific, Waltham, MA, USA). Samples were stored at -80 °C until cDNA synthesis. From each sample 1000 ng of total RNA was reverse transcribed with Sensifast cDNA Synthesis Kit (Bioline, London, UK). QPCR using SensiFAST SYBR Hi-ROX Kit with SYBR Green primers (Supplementary Table S1) were carried out on an ABI 7900 real-time PCR instrument (Thermo Fisher Scientific, Waltham, MA, USA) according to the manufacturer’s instructions. Real-time PCR results were calculated according to the following formula: relative expression level = 2^−∆Ct^, where ∆Ct = Ct (of gene of interest) − Ct (of housekeeping gene) with HPRT as an endogenous control.

### 4.5. EV Isolation and Characterization

EVs were isolated from the supernatant (SN) of BeWo, BeWo GFP transfected and BeWo HSPE1 KO cells cultured in 75 cm^2^ Tc treated flasks (Eppendorf AG, Hamburg, Germany) for 24 h in FBS free cell culture media using differential centrifugation. As a first step to remove the cell debris, the SN was centrifuged at 800 *g* for 5 min at RT. Sedimentation of apoptotic bodies at 2000 *g* for 20 min at 16 °C was followed by the centrifugation of intermediate-sized EVs (iEV) at 12,500 *g* for 20 min at 16 °C. After washing iEVs small-sized EVs (sEV) were isolated by ultracentrifugation (100,000 *g,* 70 min, 4 °C). The characterization of EVs followed the latest recommendation of the International Society for Extracellular vesicles [31]. The proteomic, miRNA and dsDNA content of EV fractions were assessed with Qubit 4 Fluorometer using the Qubit™ Protein Assay Kit, Qubit microRNA Assay Kit and Qubit 1X dsDNA HS Assay Kit, respectively. The iEV number was quantified by annexin V staining, by flow cytometry as previously described by us [32] (Supplementary Figure S5). 

Protein cargo of EVs was identified by mass spectrometry. Briefly, iEVs and sEVs were resuspended in HPLC water and subjected to repeated freeze-thaw cycles and digested in solution. The resulting peptides were cleaned using PierceTM C18 spin columns (Thermo Fisher Scientific, Waltham, MA, USA) and analyzed using a Waters nanoAcquity UPLC or a Dionex Ultimate 3000 RSLCnano system coupled to a Bruker Maxis II Q-TOF mass spectrometer (Bruker, Bremen, Germany) with CaptiveSpray nanoBooster ionization source. Following trapping, peptides were separated on a 25 cm Waters Peptide BEH C18 nanoACQUITY 1.7 µm particle size UPLC column using gradient elution. Data processing was performed with ProteinScape 3.0 software (Bruker Daltonik GmbH, Bremen, Germany). Proteins were identified using Mascot (version Mascot 2.5; Matrix Science, London, UK) search engine against the Swissprot database (2015_08). The following parameters were used: *Homo sapiens* taxonomy, trypsin enzyme, 7 ppm peptide mass tolerance, 0.05 Da fragment mass tolerance, 2 missed cleavages. Carbamidomethylation was set as fixed modification, while deamidation (NQ) and oxidation (M) as variable modifications. To identify key networks, genome ontology (GO) term enrichment was performed on the identified proteins. For gene ontology (GO) and pathway analysis, FunRich software (Uniprot and Funrich databases) was used [33].

The expression of HSPE1 in iEVs was evaluated by a flow cytometry. For exofacial labelling, isolated iEV fractions were resuspended in 100 µL of 0.2 µm filtered PBS and were stained with 0.5 µL of rabbit anti-human HSPE1 monoclonal antibody (Clone: EPR4475, Abcam, Cambridge, UK) for 20 min on ice in dark. After washing the samples with 1mL filtered PBS, samples were centrifuged (12,500 *g,* 15 min, 16 °C) and the supernatant was discarded completely. iEV pellets were labelled with 1 µL of donkey anti-rabbit IgG-Phycoerythrin antibody (20 min, on ice, in the dark). After washing iEV pellets were resuspended in 200 µL filtered PBS and analyzed on FACSCalibur flow cytometer. For intravesicular labelling first isolated iEVs were fixed and permeabilized with 2% paraformaldehyde for 10 min at room temperature on ice. Then the same procedure as for exofacial labeling was used (to distinguish the iEVs from dye-aggregates we used the same staining procedure and dye concentration without EV sample). Gating strategy is presented in Supplementary Figure S6. 

To reveal the detailed miRNA content of EVs next-generation miRNA sequencing approach was applied. BeWo-iEVs were isolated and resuspended in 12 µL of DNase, RNase free water. RNA was isolated with exoRNeasy Serum/Plasma Midi Kit (Qiagen, Venlo, The Netherlands). The RNA integrity was evaluated with Agilent Tapestation and samples with RIN values ≥7 were further processed. For library preparation, multiplex Small RNA Library Prep Kit was used according to the manufacturer’s instructions. The sequencing was performed on an Illumina MiSeq instrument and the paired-end read value was > 20 million/sample. Next, we aligned the transcriptome sequence reads to the reference genome (Ensembl GRCh38 release) with STAR version 2.5.3a using two-pass alignment mode. The mirBase annotation was used in both mapping and read counting. After alignment, the reads were associated with known miRNAs and the number of reads assigned to each miRNA was counted using ‘featureCounts’ from R package ‘Rsubread’. The data were normalized using the TMM normalization method of the edgeR R/Bioconductor package (R version 3.4.1, Bioconductor version 3.5). After pre-processing and quality control we analyzed the immunotolerance associated miRNA and trophoblast specific miRNA content with Funrich 3.1.3 [33], g:Profiler [34], and NetworkAnalyst 3.0 [35].

### 4.6. In Vitro T_reg_ Differentiation and T_reg_ Analysis

Frozen PBMCs were thawed and cultured for 1 day in 75 cm^2^ flask in RPMI-1640 cell culture media containing 12 mM L-glutamine, 10% GIBCO^®^ FBS (Thermo Fisher Scientific, Waltham, MA, USA), 100 U/mL penicillin and 100 µg/mL streptomycin. After 1 day, PBMCs were filtered through 70 µm pore sized MACS SmartStrainers (Miltenyi, Biotec GmbH, Bergisch Gladbach, Germany) and centrifuged at 800 *g* for 5 min at RT and resuspended in 100 µL of autoMACS Running Buffer (Miltenyi, Biotec GmbH, Bergisch Gladbach, Germany). PBMCs were incubated with 20 µL of anti-human CD4-Phycoerythrin conjugated antibody (Sony Biotechnology, Surrey, UK) for 20 min at RT on ice in dark. After staining, 5 mL of autoMACS Running Buffer was added and the cells were centrifuged at 800 *g* for 5 min at RT. Cells were resuspended in 1 mL of autoMACS Buffer and sorted on a SONY SH800S cell sorter (Sony Biotechnology, Surrey, UK) in 15 mL sterile tubes. The sorting efficiency was 97%. 5 × 10^5^ CD4^+^ cells were pipetted in a 12 well plate in 2mL of cell culture media. CD4^+^ T cells were supplemented with 32 IU/mL recombinant human IL-2 (Thermo Fisher Scientific, Waltham, MA, USA) and activated with bead-to-cell ratio of 1:1 of Dynabeads™ Human T-Activator CD3/CD28 for T Cell Expansion and Activation (Thermo Fisher Scientific, Waltham, MA, USA). Treatment of CD4^+^ T cells with rHSPE1 in different concentrations (1 µg/mL, 2.5 µg/mL, 10 µg/mL, 15 µg/mL) or 1 × 10^6^ annexin V positive BeWo GFP transfected- derived iEVs or BeWo HSPE1 KO-derived iEVs was given. After 72 h activation, Dynabeads were removed and cells were washed (800 *g*, 5 min, RT) and stained with anti-human CD45RO-Phycoerythrin, anti-human CD25-Fluorescein isothiocyanate (FITC), anti-human CD3- Alexa Fluor^®^ 647, and anti-human CD127-Phycoerythrin/Cyanine7 monoclonal antibodies in one tube and anti-human HLA-DR Phycoerythrin/Cyanine7 together with anti-human CD25 FITC in another FACS tube for immunophenotyping and sorting of regulatory T cells (for gating strategy, see Supplementary Figure S6, for used antibodies see Supplementary Table S2). FACS data were analyzed with Flowjo v10.3 (Tree Star Inc., OR, USA) software. 

### 4.7. Holomicroscopy of T_reg_ Cells

CD25^hi+^ CD127^lo+^ sorted T_reg_ cells were characterized by holomicroscopy (HoloMonitor M4 Phase Holographic Imaging, Lund, Sweden). Five thousand T_reg_ cells were resuspended in 500 µL of cell culture media and seeded on µ-Slide 8-Well with ibiTreat (Ibidi, Martinsried, Germany) and incubated for 30 min to allow cell adhesion. Cell migration (the shortest direct distance from the starting point to the end point (μm)) and motility (the actual way traveled from the starting point to the end point (μm)) were monitored for 3 h with 60 sec time-lapse intervals. Time-lapse automatic background thresholding method was used with the minimum error sets algorithm (adjustment = 128) for evaluation of images. The total number of evaluated images were 180 and the number of evaluated cells per image was 10. For manual cell identification, objects of the marginal zone were eliminated. HoloStudio M4 2.5 program was used to analyze data.

### 4.8. Single-Cell Transcriptomics Analysis

Regulatory T cells, CD4^+^ T cells and 68K PBMCs datasets were downloaded from 10x Genomics datasets available on link. Phyton based Scanpy version 1.3.7 was used for pre-processing and data analysis. For data filtering and quality control the following parameters were used: cell filter <= 200 genes (filtered out cells that have less than 200 genes expressed), genes filter <3 (filter out genes that are detected in less than 3 cells) and mitochondrial filter <0.05 (filter out cells that have more than 5% of mitochondrial reads). Afterwards, the highly variable genes (HVG) were identified in the datasets. Cell were clustered according to the Louvain algorithm (resolution = 0.8). Data were visualized as Uniform Manifold Approximation and Projection (UMAP). Cell clusters were annotated manually, using the top 50 genes expressed in each cell cluster and based on the expression of known cell subtype markers from the literature. 

### 4.9. Statistics

GraphPad Prism version 7.0 (GraphPad Software, La Jolla California, USA) was used for statistical analysis. Two-sided Student’s unpaired *t*-test was used for normally distributed data, and ANOVA analysis followed by Bonferroni correction was applied for multiple parameter analysis. In the cases of non-normal distribution data, Mann–Whitney U or Wilcoxon tests were used, while multiple parameter analysis was calculated by ANOVA test followed by Kruskal–Wallis test. The level of significance was set at *p* < 0.05.

The datasets generated during and/or analyzed during the current study are available from the corresponding author on reasonable request.

## 5. Conclusions

EVs, as communication modules between trophoblast and maternal immune cells, represent a continuous dynamic bridge between the mother and the fetus and define the differentiation and polarization of maternal immune cells during pregnancy. We presented a protein-miRNA hybrid molecular pattern, highlighting the HSPE1 protein, of trophoblastic-derived extracellular vesicles, that is highly potent orchestrator of T_reg_ cells differentiation.

## Supplementary Materials

Supplementary materials can be found at https://www.mdpi.com/1422-0067/20/14/3457/s1.
