# Supplementary material for: Unravelling the Role of Trophoblastic-Derived Extracellular Vesicles in Regulatory T Cell Differentiation
Source: Int J Mol Sci. 2019 Jul 14;20(14):3457. doi: 10.3390/ijms20143457 (PMC6678568; doi:10.3390/ijms20143457)
Supplement: Supplementary file 1 [file ijms-20-03457-s001.zip › ijms-543377-SI/Supplementary File 1.pdf]

Supplementary Figure 1 Mass spectrometry of small-sized EV fraction

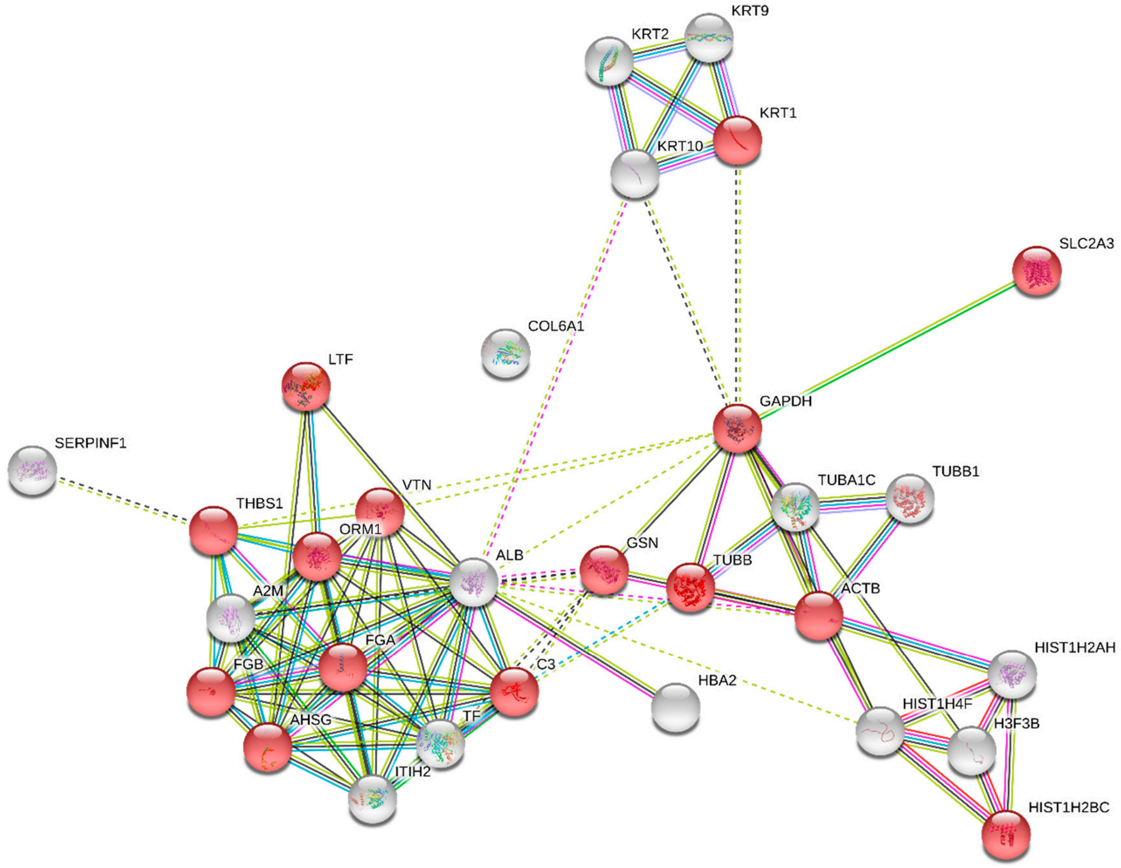

K-mean clustering of proteins identified in BeWo-derived sEVs. Red dots represented proteins are involved in immune system processes.

Supplementary Figure 2 Functional integrity of T_{reg} cells determined by holomicroscopic analysis

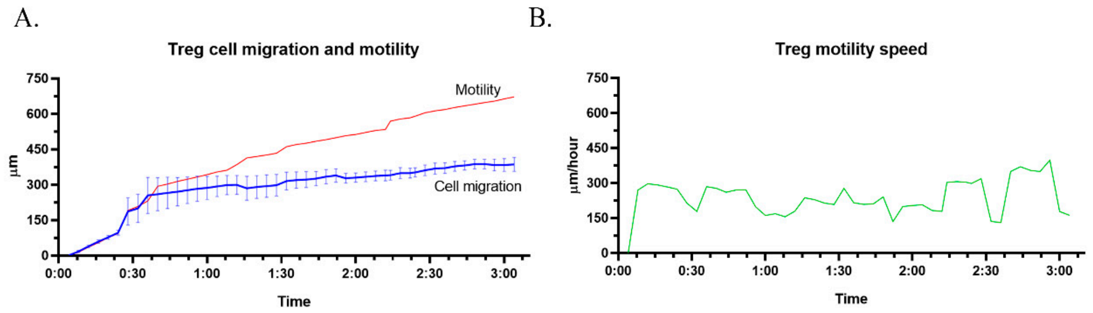

Holomicroscopic analysis of sorted CD25+CD127lo T_{reg} shows cell migration and motility (A), and the speed of motility (B).

Supplementary Figure 3 Optimization of transfection

A.

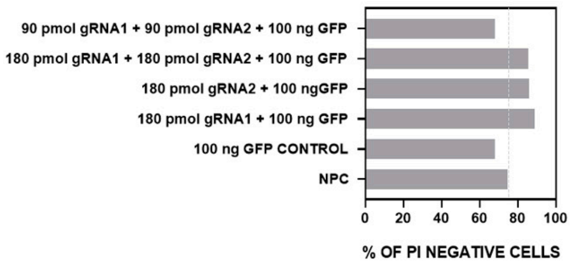

B.

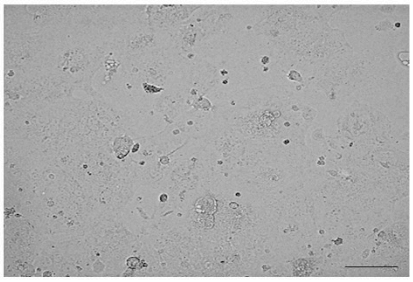

C.

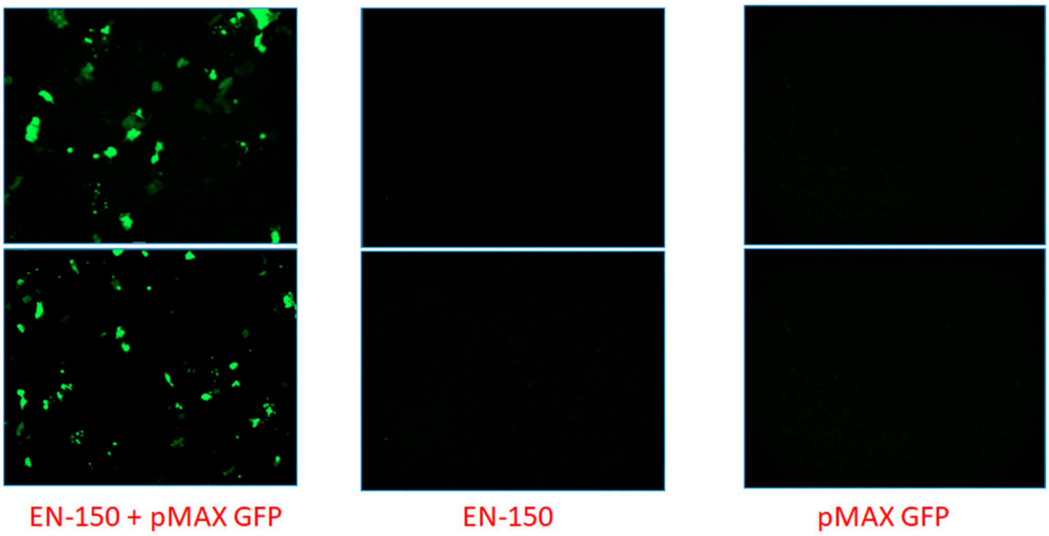

A. Percentage of living cells (PI negative cells) 24 hours post-transfection with different gRNAs (sgRNA-1 G*C*U*GCUGAAACUGUAACCAA and sgRNA-2 U*A*A*ACGCUUGUCCUGCCUGU) and gRNA concentration. B. Microscopic evaluation of BeWo cell transfected with gRNA2 after 10 days. C. Validation of optimal program (EN-150) selection.

Supplementary Figure 4 Generation of HSPE1 gene knockdown by CRISPR-Cas9 sgRNP

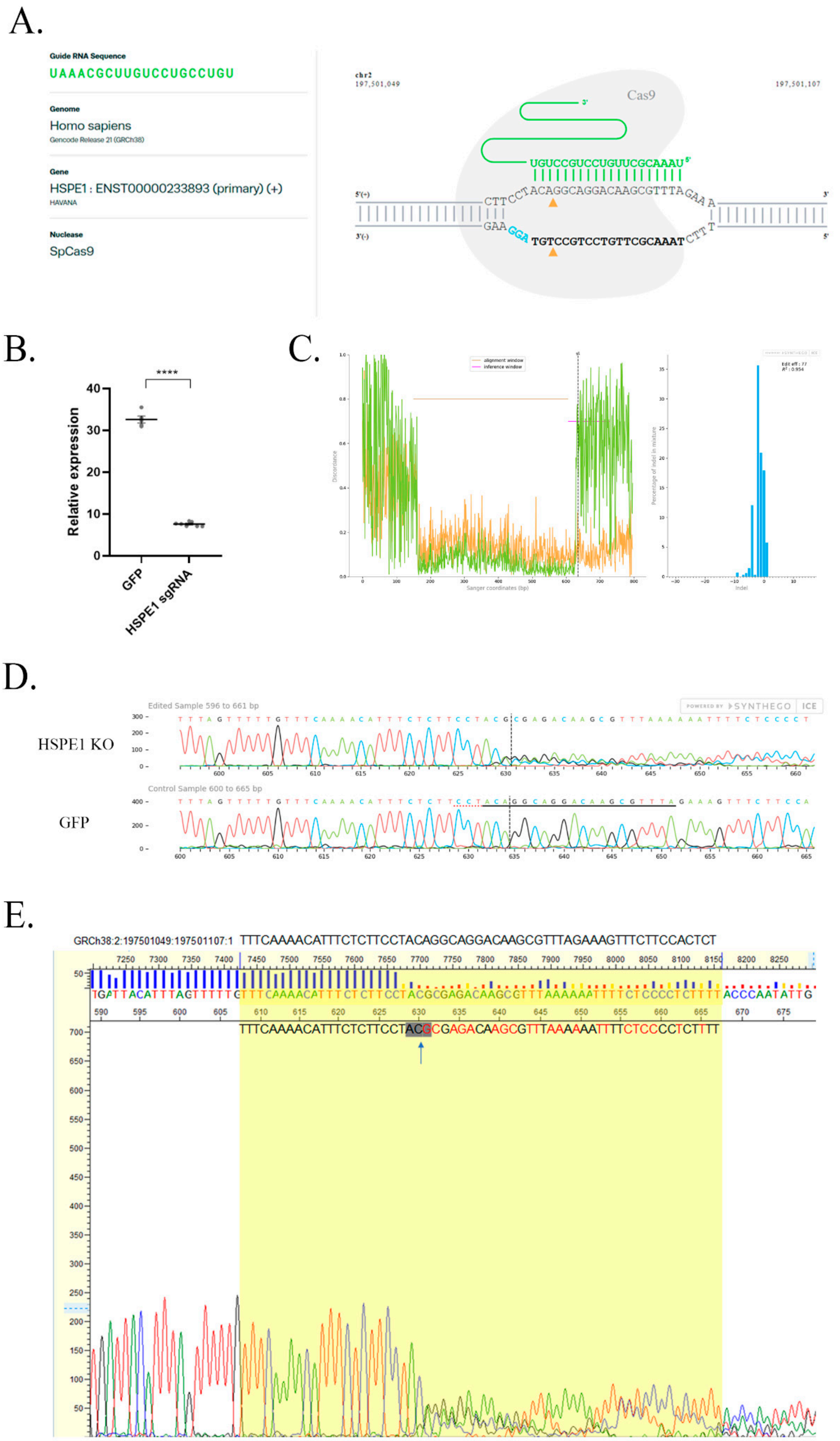

A. *HSPE1* specfic gRNA design used to transfect BeWo cells. B. qPCR validation of transfection. C. ICE analysis based on Sanger sequencing validation to assess transfection efficiency. D. Sanger sequencing validation of transfection. E. Showing the targeted indel region by gRNA and the corresponding electrophoretogram.

Supplementary Figure 5 Characterization of BeWo-derived EVs

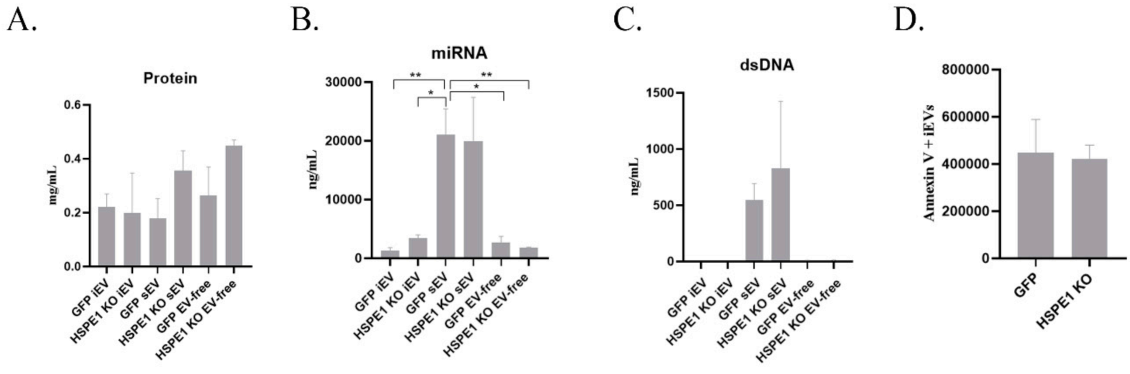

A. Protein content of GFP transfected (mock transfection) and HSPE1 KOEVs. B. miRNA content of GFP transfected and HSPE1 KO-EVs. C. dsDNA content of GFP transfected and HSPE1 KO-EVs D. Annexin V+ iEVs isolated from GFP transfected and HSPE1 KO BeWo cells.

Supplementary Figure 6 Gating Strategy

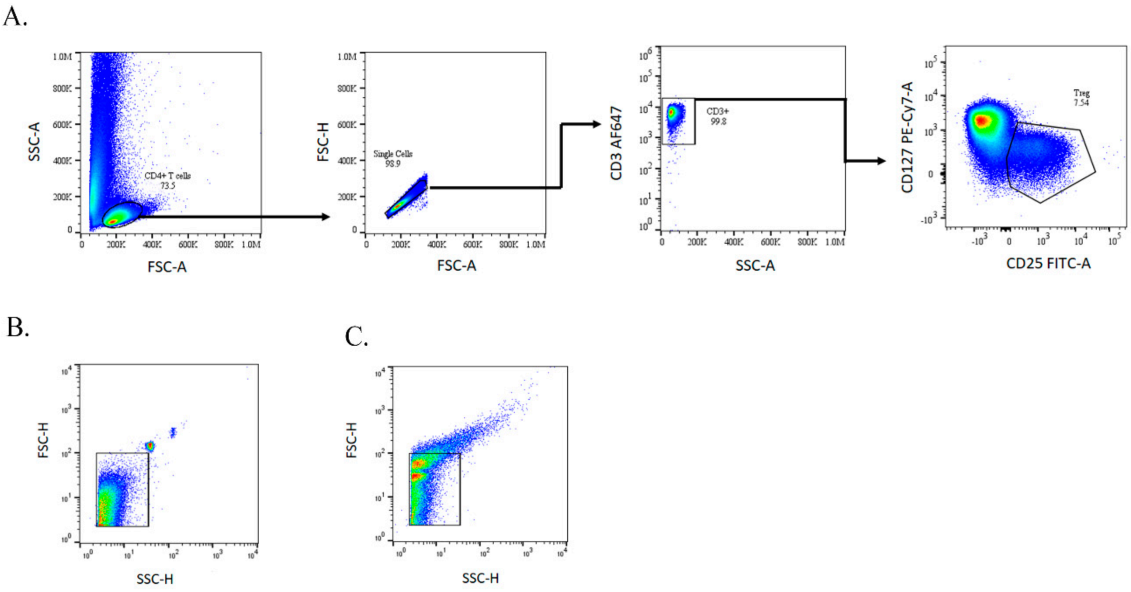

A. Gating strategy for the analysis of regulatory T cells. The morphologically intact cells were selected on the basis of FSC/SSC dot plot. Singlets were determined on FSC-A/FSC-H dot plot. CD3 positivity was used to assess purity of CD4+ sorted T cells. CD25+ CD127lo immune phenotype was used to set the gate for T_{reg} cells. B. iEV gating strategy: upper corner of the gate is below the 1 µm silica beads. C. Megamix beads were used to detect the lower threshold of the iEV gate (the smallest size were the 200 nm beads). FSC: forward scatter, SSC: side scatter, H: height, A: area

Supplementary Table 1

GENE	FORWARD PRIMER<br>OLIGO SEQUENCE (5' TO 3')	REVERSE PRIMER<br>OLIGO SEQUENCE (5' TO 3')
**HPRT**	GGTCAGGCAGTATAATCCAAAG	GTCAAGGGCATATCCTACAAC
**HSPE1**<br>**(for qPCR)**	GGTTGAAAGGAGTGCTGCTGAA	GAATGGGCAGCATCATGTTGAT
**HSPE1**<br>**(for sanger sequencing)**	TAGAGCAGAGTACGAGTCTGAG	CCTTTCCTTTAGAACCCGATCC

Supplementary Table 2

Antibody/Dye name	Clone*	Manufacturer	Catalogue number	Applied Methods
PE/Cy7 anti-human CD127 (IL-7Rα)	A019D5	SONY	2356595	FACS
Alexa Fluor® 647 anti-human CD3	SK7	SONY	2324125	FACS
PE/Cy7 Mouse IgG2b, κ Isotype Ctrl	MPC11	SONY	2601625	FACS
FITC Mouse Anti-Human CD25	M-A251	BD Biosciences	555431	FACS
Anti-human HSPE1 rabbit Ab	EPR4475	Abcam	ab109489	FACS; immunoblot
Anti-rabbit IgG1 κ1 – AF488		Abcam	ab150077	FACS
FITC Annexin V		Biolegend	640906	FACS
